# Population genetics and phylogeography of alfalfa mosaic virus in China and a comparison with other regional epidemics based on the *cp* gene

**DOI:** 10.3389/fpls.2022.1105198

**Published:** 2023-02-14

**Authors:** Xin Wang, Chenchen Liu, Zhaoyan Tan, Jiantai Zhang, Rongqun Wang, Yuanhong Wang, Xiliang Jiang, Beilei Wu

**Affiliations:** ^1^ Institute of Plant Protection, Chinese Academy of Agricultural Sciences, Beijing, China; ^2^ Department of Plant Protection, College of Horticulture and Landscape Architecture, Tianjin Agricultural University, Tianjin, China; ^3^ People's Congress Standing Committee Office, Xiuzhou District, Jiaxing, Zhejiang, China

**Keywords:** AMV, Bayesian methods, epidemiology, molecular population genetics, phylogenetics, virus evolution

## Abstract

Alfalfa mosaic virus (AMV) is the most pervasive epidemic virus affecting alfalfa production. However, detailed investigations on the molecular population genetics and evolutionary dynamics of AMV are scarce. This study aimed to report on a large-scale long-term survey of genetic variability in AMV populations from China and perform a comparative analysis of AMV population genetics in the three most thoroughly studied countries to date: China, Iran, and Spain. The study was based on the analysis of the coat protein gene (*cp*) using two analytical approaches: an analysis of molecular variance (AMOVA) and a Bayesian Markov Chain Monte Carlo approach that investigates the association between geographical origin and phylogeny. Both analytical approaches found significant genetic differentiation within localities, but not among localities nor among provinces. This observation might result from inappropriate agronomical practices involving extensive exchange of plant materials followed by rapid viral diversification within localities. In the Chinese population, both methods found that genetic diversification in AMV was strongly associated with different bioclimatic zones. Rates of molecular evolution were similar in the three countries. The estimated epidemic exponential population size and growth rate suggest that the epidemics grew faster and with higher incidence in Iran, followed by Spain and China. Estimates of the time to the most recent common ancestors suggest that AMV was first seen in Spain by the beginning of the twentieth century and later on in eastern and central Eurasia. After ruling out the existence of recombination breakpoints within the *cp* gene, a codon-based selection analysis per population was performed and identified many codons under significant negative selection and a few under significant positive selection; the latter varied among countries, suggesting regional differences in selective pressures.

## 1 Introduction

*Medicago sativa* L. (alfalfa, syn. lucerne) is the most important forage legume globally (Michaud et al., 1988), owing to its adaptability to drought conditions, ability to form symbiosis with rhizobia and fix atmospheric nitrogen (Carlsson and Huss-Danell, 2003), high protein value (Howarth et al., 1977; Vance et al., 1979; Ruckle et al., 2017), and yield potential with ‘cut and carry’ cropping regimens (Small, 1996). The area of alfalfa planting in China currently ranks second in the world. However, the continuous increase of alfalfa planting areas and the accumulation of cropping and spreading vectors has led to an increased frequency of outbreaks of alfalfa virus diseases. The more common symptoms range from dwarfed plants, shrinking and yellowing of leaves, lower yield, and quality of herbage (Xu and Nie, 2006; Liang et al., 2017). Among all alfalfa diseases, alfalfa mosaic virus disease (AMVD), whose etiological agent is the alfalfa mosaic virus (AMV; species alfalfa mosaic virus, genus *Alfamovirus*, family *Bromoviridae*), is the most serious and prevalent one in China (Wang et al., 2021).

AMV was first identified by Weimer in 1931 (Hull, 1969). Many studies have provided information on the morphology, physicochemical properties, regulation of gene expression, and host range of AMV through isolation and purification of the virus (Kasteel et al., 1997; Sánchez-Navarro and Bol, 2001; Sánchez-Navarro et al., 2006; Xu and Nie, 2006). AMV has a wide host range and is distributed worldwide (Kvícala, 1975; Garran and Gibbs, 1982; Popp et al., 2000; Mallor et al., 2002; Esfandiari et al., 2005; Liang et al., 2017; Wang et al., 2021). In nature, AMV infects approximately 150 species from 22 botanical families; however, when experimental susceptible hosts are included, the number of susceptible species expands to over 600 in 70 families (Jaspars and Bos, 1980; Bol, 2008; Liang et al., 2017).

The whole genome of AMV consists of three positive-stranded RNAs. Monocistronic RNAs 1 and 2 encode the nonstructural P1 and P2 proteins to form the RNA-dependent RNA polymerase (RdRP). The dicistronic RNA 3 encodes the movement (MP) and coat (CP) proteins, the latter translated from a subgenomic RNA 4 (Sánchez-Navarro and Bol, 2001). RNA 3 can be transported from cell to cell by both tubule-forming and non-tubule-forming MPs if a specific MP–CP interaction occurs (Sánchez-Navarro et al., 2006), and CP is involved in cell-to-cell movement (Van der Vossen et al., 1994; Kasteel et al., 1997) without requiring the formation of stable virus particles (Sánchez-Navarro and Bol, 2001). This abundance of molecular information is at odds with the number of studies tackling AMV population genetic structure, molecular epidemiology, and phylogeography. Early work by Parrella et al. (2000) and Xu and Nie (2006) suggested that the AMV global population could be divided into different groups according to geographic regions, but the amount of data used in these studies was limited and this jeopardized the generality and validity of the resulting conclusions. Bergua et al. (2014) collected and characterized 60 isolates of AMV from Spain and highlighted the rich genetic diversity of AMV populations, with recombination playing an important role (Bergua et al., 2014).

This study aimed to address the population genetics of AMV in China, using the sequence of the *cp* cistron from 76 new isolates (**Supplementary Table S1**). In addition, isolates were collected on a global scale (232 isolates comprising 86 isolates from China, 55 isolates from Iran, and 91 isolates from Spain) (**Supplementary Tables S1**, **S2**), and the divergence time and geographic origin were explored using Bayesian and analysis of molecular variance (AMOVA) methods. Moreover, the Chinese population was thoroughly investigated, including (1) the rates of migration among locations and the effective epidemic size, (2) the influence of selection and genetic drift, and (3) the time since the first introduction of the virus in China. Subsequently, to assess whether the characteristics described for the Chinese populations were unique or shared by other well-documented AMV epidemics, similar analyses were performed with previously published *cp* sequences from Spain (56) and Iran (91). The comparison among these three epidemics has allowed the determination of some general principles driving the epidemiology and evolution of AMV.

## 2 Materials and methods

### 2.1 Collection of AMV isolates from China

A total of 516 alfalfa samples was collected from 24 locations of 13 major alfalfa-producing provinces and four climatic regions in China from 2016–2019. The isolate names, their hosts, dates and sites of collection, and climatic regions are shown in **Supplementary Table S1**. Positivity of samples for AMV infection was determined by RT-PCR using the primers shown in **Supplementary Table S3**. The primers used for amplification of the AMV *cp* gene fragment by RT-PCR are listed in **Supplementary Table S4**.

### 2.2 Sequencing of *cp* gene of AMV isolates from China and published data from other locations

#### 2.2.1 Cloning and sequencing of cp gene

Total RNA was extracted from alfalfa leaves systemically infected by AMV. The extracted RNA was used as a template for RT-PCR amplification in a 20-μL reaction containing total RNA (50–5000 ng), 1 μL gDNA Remover, 1 μL primer AMV-R (**Supplementary Table S4**), 10 μL of 2× Reaction Mix, 1 μL Enzyme Mix with 5 mM dNTP (each), and RNase-free water to 20 µL. The reaction was incubated at 25°C for 10 min, 42°C for 45 min, and 85°C for 5 s, then the PCR commenced with the system of 2× EasyTaq^®^ PCR Super Mix (+ dye) 25 µL, cDNA 2 µL, pimer *cp*-F 1 µL, primer *cp*-R 1 µL, and 21 µL of ddH_2_O to total 50 µL (TransGen, China). PCR cycling conditions comprised an initial denaturation step at 94°C for 3 min, 35 cycles of denaturation at 94°C for 1 min, annealing at 57°C for 1 min, and extension at 72°C for 3 min, followed by a final extension at 72°C for 5 min. The expected RT-PCR products of the *cp* gene were 666 bp, using primer pairs *cp* F/*cp* R (**Supplementary Table S5**), and together covered the entire length of the viral genome. The PCR product segments were electrophoresed in 1.0% agarose gels and purified by the BioTeq PCR quick Gel Extraction Kit (BioTeq, USA). The purified fragments were cloned into the pMD18-T vector (Takara, Dalian, China) and used to transform *Escherichia coli* JM110. Insert sequences were determined for at least three clones for each fragment using either the ABI (ABI BigDye 3.1, Applied Biosystems) or Beckman (GeXP with Genome Lab DTCS sequencing kit) system. Sequence data were assembled using DNASIS version 3.5 (Hitachi, Tokyo, Japan), Laser gene (DNASTAR, Madison WI, USA), or BIOEDIT version 5.0.9 (Hall, 1999).

The published *cp* gene sequences of AMV isolates from Iran, Spain, and other countries were collected from the GenBank database (**Supplementary Table S1**).

### 2.3 Computational analyses for the populations of AMV

#### 2.3.1 Phylogenetic analyses

All of the *cp* genes of AMV populations from China, Iran, and Spain, respectively, were aligned with MUSCLE (Edgar, 2004) as implemented in MEGA version 5.0 (Tamura et al., 2011). The best model of nucleotide substitution was determined by MODELTEST version 3.7 (Posada and Crandall, 2001). SplitsTree version 4 was used for the split-decomposition network analysis (Huson and Bryant, 2006). Maximum credibility clade (MCC) phylogenetic reconstructions were conducted using BEAST version 1.5.4 (Drummond and Rambaut, 2007). Markov Chain Monte Carlo (MCMC) simulations were run for 10^7^ generations to ensure convergence of all parameters. Branches with a posterior support probability of 0.50 were collapsed.

#### 2.3.2 Population genetics analyses

Population genetics analyses and diversity measures for all populations (Tajima’s *D*, Ewans-Watterson, Chakraborty, and Fu’s *F*) were calculated by ARLEQUIN (http://cmpg.unibe.ch/software/ arlequin3) (Excoffier et al., 2005). The best alignment of the sequences of every population was inputted into the ARLEQUIN version 3.0 and the corresponding analysis was performed.

#### 2.3.3 Inferring selection patterns

Selective pressures operating at each codon were evaluated based on the difference between synonymous (*d_S_*) and nonsynonymous (*d_N_*) substitution rates for the *cp* gene calculated by MEGA X (Tamura et al., 2011). As a first approach, values of *d_N_*−*d_S_ <*0, = 0, or >0 indicate purifying selection, neutral evolution, and positive selection, respectively.

#### 2.3.4 Phylogeographic analyses

Data were divided into different groups among bioclimatic regions (**Supplementary Figure S1**). To determine the extent of geographic structure in AMV populations, BaTS version 1.0b2 (Parker et al., 2008) was used to compute the parsimony score (*PS*) (Slatkin and Maddison, 1989), the association index (*AI*) (Wang et al., 2001), and the maximum monophyletic clade size (*MC*) (Parker et al., 2008), and to assess the significance of these three statistics. The first 10% of sampled trees were discarded as burn-in and 104 randomizations were performed to estimate the null distributions of the three statistics.

#### 2.3.5 Recombination analyses

The data sets from the three countries were analyzed respectively for the recombination using the methods of RDP, BOOTSCAN, CHIMERA, GENECONV, LARD, MAXCHI, SISCAN and 3SEQ implemented in RDP4 in the default configuration (Martin et al., 2010) as well as the GARD and PHI test in SplitsTree (Huson and Bryant, 2006). Only those recombination events predicted by at least five of these methods and with a *p* value < 0.05 were regarded as valid.

## 3 Results

### 3.1 Incidence of AMV infection in Chinese alfalfa-producing regions

A total of 516 alfalfa samples was collected from 23 locations in 13 provinces distributed along the main alfalfa-producing Chinese provinces between 2016 and 2019. These provinces encompassed four climatic regions (plateau mountain climate, temperate continental climate, temperate monsoon climate, and subtropical monsoon climate) (**Supplementary Figure 1**). All samples were tested for infection with AMV using a specific pair of primers that amplify the *cp* cistron. Across the sampling period, the incidence of AMV was estimated to be as high as 57.6% (95% adjusted Wald confidence interval: 52.3%–60.8%). RT-PCR and sequencing yielded 76 complete sequences of the AMV *cp* gene from the 23 locations.

### 3.2 Population genetics analyses of AMV in China

#### 3.2.1 Parsimony network and MCC tree

The parsimony networks revealed the existence of two major clades, one more divergent (Clade III) and one less so (Clades I and II) (**Supplementary Figure S2**), which was consistent with the clustering identified by the MCC tree obtained with BEAST (**Supplementary Figure S3**). In the MCC tree, Clade A (yellow) contained isolates from three climatic regions (plateau mountain climate, temperate continental climate, temperate monsoon climate), while Clade B (blue) comprised a single isolate from Inner Mongolia, and Clade C (purple) contained isolates from all of the climatic regions. Clade I in **Supplementary Figure S2** corresponded with Clade A in **Supplementary Figure S3**, and Clades II and III were consistent with Clades B and C, respectively. The software package RDP 4, as well as GARD and PHI tests in SplitsTree showed no evidence of recombination, therefore migration among populations would explain the reticulated pattern.

**Figure 1:**
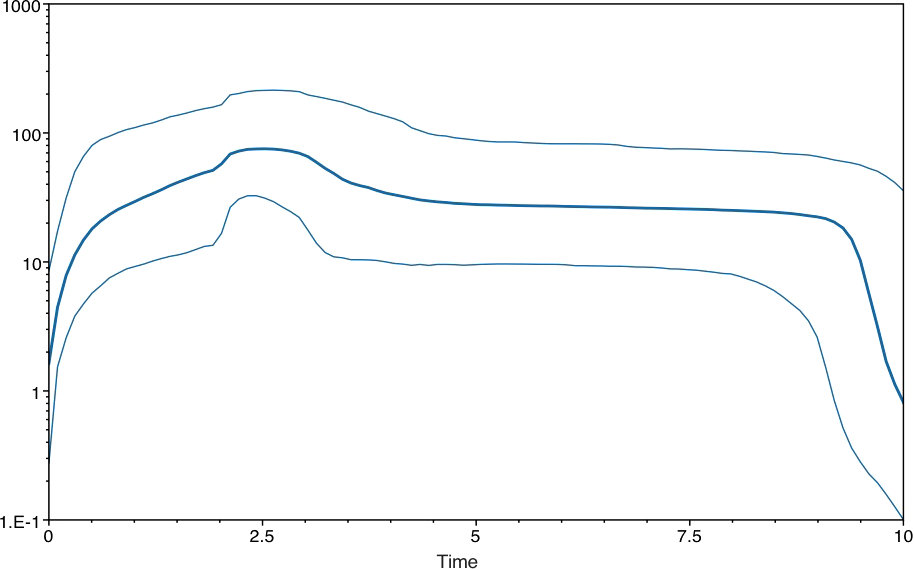
Plots of the effective number of infections *vs* time in the Chinese AMV population by skyline in the default configuration of BEAST.

To explore the possible existence of a molecular clock, TempEst (Rambaut et al., 2016) was employed, and the algorithm found a significant correlation between genetic divergence and time (**Supplementary Figure S4**). The relevant parameter estimates from the Bayesian analysis were time to most recent common ancestor (TMRCA)=1951.2 ± 2.5 years ago; an effective number of infections of 114.0±6.6; an exponential growth rate of 0.023 ± 0.001 per year; and a rate of molecular evolution of 9.1 ± 0.4×10^−4^ substitutions/site/year (**Table 1**). The plots of the effective number of infections versus time are shown in **Figure 1**.

**Table 1:** Relevant estimates from the Bayesian analysis for the Chinese AMV population.

	Mean	SEM	Median	Lower 95% HPD	Higher 95% HPD
TMRCA	1951.1613	2.5164	1957.2309	1991.7844	1896.3123
Exponential population size	114.027	6.6308	100.1071	30.6142	231.9648
Exponential growth rate	0.0228	7.86E-04	0.0221	-1.19E-02	0.0624
Mean rate of evolution	9.09E-04	4.34E-05	8.68E-04	3.78E-04	1.55E-03

The BaTS algorithm was run to explore whether the MCC clustering was significantly associated to (i) geographic origin, (ii) host species, and (iii) bioclimatic zone. Firstly, using geographic origin as a trait, a highly significant association was found (*AI* = 8.8954, *P <*0.0001; *PS* = 69.3392, *P <*0.0001) (**Table 2**). In the single collection sites of Cangzhou (*MC* = 1.996, *P* = 0.02), Dali (*MC* = 1.9891, *P* = 0.036), Qiqihaer (*MC* = 1.6233, *P* = 0.015), Shihezi (*MC* = 2, *P* = 0.013), and Hangzhuo (*MC* = 2, *P* = 0.005) (**Table 2**), the isolates were predominantly driving the differentiation. Based on the provinces (*AI* = 5.99, *P <*0.0001; *PS* = 52.3072, *P <*0.0001) (**Table 3**), significant differences were found among Anhui, Hebei, Yunnan, Guizhou, Zhejiang, Heilongjiang, Tibet, Qinghai, Ningxia, Shanxi, Shannxi, Inner Mongolia, and Xinjiang. Secondly, using host species (*M. sativa*, *G. pentaphyllum*, *N. tabacum*, *N. glutinosa, C. japonica*, *J. procumbens*, *V. persica*, and *T. pretense*) as a discrete trait, no significant associations were found (**Table 4**). Viruses isolated from members of the family *Solanaceae* clustered together (*MC* = 2, *P* = 0.005), while viruses isolated from other families were well mixed in the MCC tree (**Table 5**). Finally, significant associations were found with bioclimatic zones (*AI* = 3.9158, *P <*0.0001; *PS* = 37.053, *P <*0.0001) (**Table 6**). Furthermore, within the subtropical monsoon zone, a significant difference was found among locations (*MC* = 4.0014, *P <*0.0035) (**Table 6**).

**Table 2:** BaTS algorithm analysis for Chinese AMV population based on geographic locations.

	Mean	SEM	Median	Lower 95% HPD	Higher 95% HPD	*p*
AI	8.8954			8.0615	9.7097	<0.0001
PS	69.3392			67	71	<0.0001
MC (Bengbu)						
MC (Cangzhou)	1.996			2	2	0.02
MC (Dali)	1.9891			2	2	0.036
MC (Guiyang)						
MC (Hangzhou)	2			2	2	0.005
MC (Harbin)						
MC (Langfang)						
MC (Lanzhou)						
MC (Lhasa)						
MC (Linan)						
MC (Minhe)						
MC (Qiqihaer)	1.6233			1	3	0.015
MC (Rikaze)						
MC (Shihezi)	2			2	2	0.013
MC (Taiyuan)						
MC (Urumqi)						
MC (Wulan)						
MC (Wuzhong)						
MC (Xian)						
MC (Xianggelila)						
MC (Xilianhaote)						
MC (Xining)						
MC (Yangling)						
MC (Yining)						

**Table 3:** BaTS analysis for the different provinces of China.

	Mean	SEM	Median	Lower 95% HPD	Higher 95% HPD	*p*
AI	5.99			5.0688	6.713	<0.0001
PS	52.3072			50	55	<0.0001
MC (Anhui)						
MC (Hebei)						
MC (Yunnan)						
MC (Guizhou)	2.0172			2	2	0.0334
MC (Zhejiang)	4.0013			4	4	0.0001
MC (Heilongjiang)						
MC (Tibet)						
MC (Qinghai)						
MC (Ningxia)	1.8319			1	2	0.0335
MC (Shanxi)						
MC (Shaanxi)						
MC (Inner Mongolia)						
MC (Xinjiang)	1.9798			2	2	0.0067

**Table 4:** BaTS algorithm analysis for for the Chinese AMV population based on host species level.

	Mean	Lower 95% HPD	Higher 95% HPD	*p*
AI	1.17306	0.92799	1.39105	0.1360
PS	7	7	7	1.0000
MC (*M. sativa*)	14.31641	11	17	0.0740
MC (*G. pentaphyllum*)				
MC (*N. tabacum*)				
MC (*N. glutinosa*)				
MC (*C. japonica*)				
MC (*J. procumbens*)				
MC (*V. persica*)				
MC (*T. pratense*)				

**Table 5:** BaTS algorithm analysis for for the Chinese AMV population based on host species level.

	Mean	Lower 95% HPD	Higher 95% HPD	*p*
AI	0.8976	0.674	1.0571	0.0880
PS	5	5	5	0.0050
MC (*Fabaceae*)	14.3164	11	17	0.0860
MC (*Cucurbitaceae*)				
MC (*Solanaceae*)	2	2	2	0.0050
MC (*Theaceae*)				
MC (*Cupressaceae*)				
MC (*Scrophulariaceae*)				

**Table 6:** BaTS algorithm analysis for the Chinese AMV population based on the bioclimatic zones.

	Mean	SEM	Median	Lower 95% HPD	Higher 95% HPD	*p*
AI	3.9158			3.142	4.6984	<0.0001
PS	37.053			35	39	<0.0001
MC (Subtropical monsoon)	4.0014			4	4	0.0035
MC (Temperate monsoon)	2.0229			2	2	0.0645
MC (Temperate continental)	2.8548			2	4	0.0645
MC (Plateau mountain)	4.3657			4	6	0.0956

#### 3.2.2 Selection analyses

**Figure 2:**
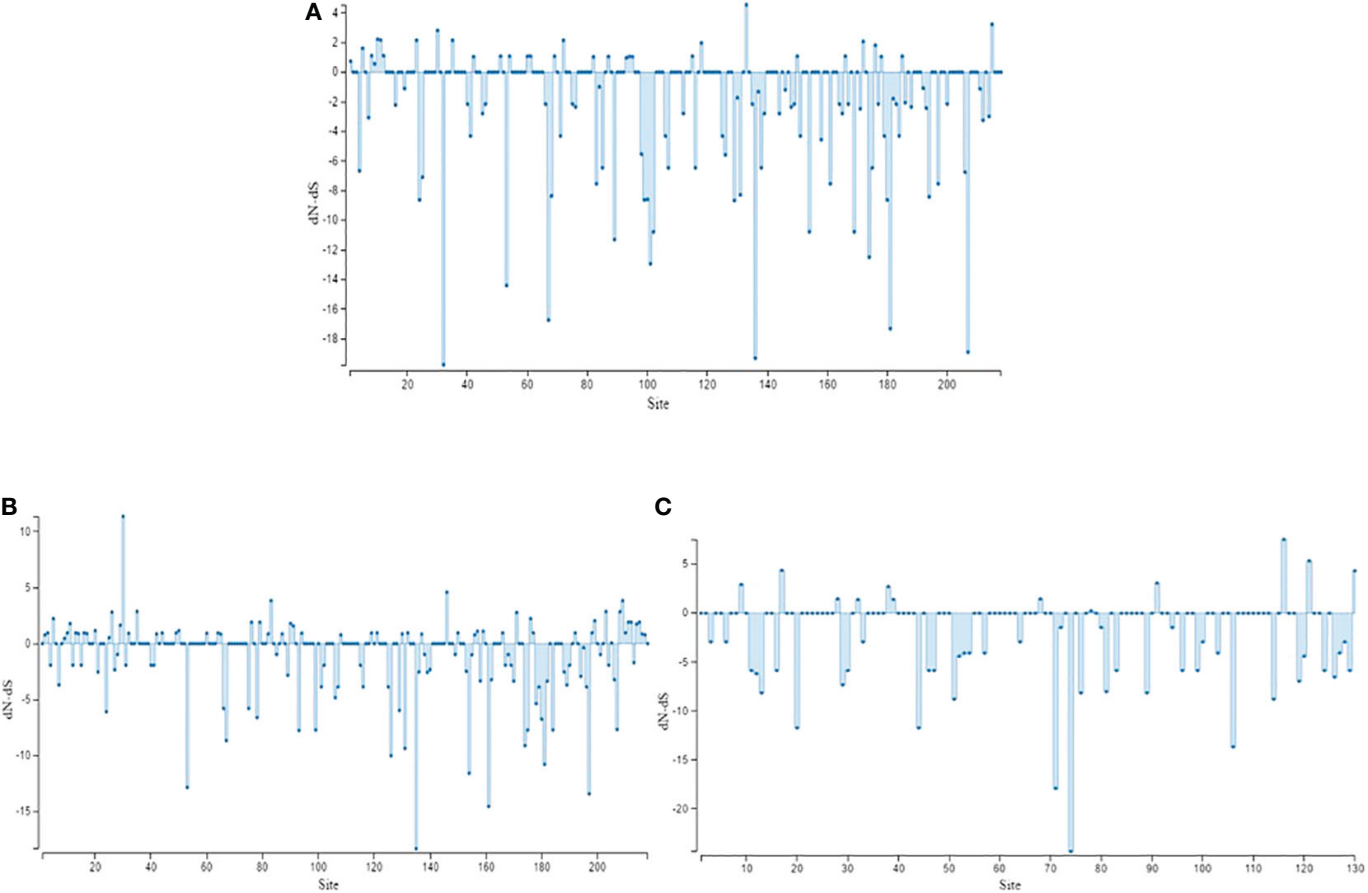
Selection analyses for AMV populations by DnaSP version 5. **(A)** Chinese population; **(B)** Iranian population; **(C)** Spanish population.

**Figure 2A** and **Supplementary Table S5** show that there are quite a lot of sites under purifying selection.

#### 3.2.3 Population structure

As a complementary approach to the association analyses conducted with BaTS, population genetics AMOVA was also performed. The data were divided into different groups based on different provinces and bioclimatic regions of China (**Supplementary Figure S1**); the bioclimatic regions of China included four groups—plateau mountain, temperate continental, temperate monsoon, and subtropical monsoon. A population genetics approach was used to further evaluate the geographic population structure suggested by BaTS. BaTS exhibited significant differences among provinces (*AI* = 5.99, *P <*0.0001; *PS* = 52.3072, *P <*0.0001) (**Table 3**), and also among localities within province (*AI* = 8.8954, *P <*0.0001; *PS* = 69.3392, *P <*0.0001) (**Table 2**), which is similar to the AMOVA results with *P* = 0.0289 (**Supplementary Table S6**). Furthermore, a significant difference was observed among different samples taken from the same locality (**Supplementary Tables S6**, **S7**). For instance, in Hangzhou in Zhejiang province, significant variance was detected among the samples (*P <*0.005) (**Table 2**). None of the neutrality tests found a significant effect of selection. For the different hosts of AMV, there was a difference only on *M. sativa*; there was no evidence of differences with the other hosts (**Table 4**). The samples from four different bioclimatic zones were also analyzed for population structure. The *P* values indicate that the virus clearly shows differences between bioclimatic regions and among localities within each of the four bioclimatic regions (**Table 5** and **Supplementary Table S7**).

### 3.3 Comparison with local epidemics in Iran and Spain

#### 3.3.1 Parsimony network MCC tree

AMV isolates from Iran and Spain were selected for population genetics analyses since they were the most thoroughly sampled isolates. For the Iranian population, the network suggests a single clade with very few divergent genotypes (**Supplementary Figure S5**), and the pattern is distinct from that of the Chinese network. Recombination among the populations from Iran, Spain, and China was not detected by PHI. The parsimony network MCC tree for isolates from Spain (**Supplementary Figure S6**) was more similar to the Chinese one, with three well-differentiated groups and the suggestion of recombination between and within the groups by the PHI test. Isolates from Zaragoza province were all over the network. Andalucian isolates were mostly homogenously grouped and the group included an isolate from Zaragoza, suggesting that an introduction from Andalucia may be the origin of the local epidemic in Zaragoza (**Supplementary Figure S6**).

TempEst found a significant correlation between genetic divergence and time for the Iranian population, allowing for the estimation of molecular evolutionary rates (**Supplementary Figure S7**). The TMRCA of the Iranian population was 1996.3±1.4 years, with an effective number of infections of 7.4±4.6×10^6^, an exponential growth rate of 0.67±0.04 per year, and a rate of molecular evolution of 8.05±0.50×10^−4^ substitutions/site/year (**Table 7**). These data suggest that the epidemic is older in Iran compared with that in China, and that it expanded at a 10-fold faster rate, with many more infections. For the Spanish population, TempEst found a significant time-stamp in genetic divergence (**Supplementary Figure S9**), allowing for the estimation of rates of evolution. The TMRCA was 1891.0±5.5 years, with an effective number of infections of 1016.9±73.1, an exponential growth rate of 0.043±0.002 per year, and a rate of molecular evolution of 2.79±0.08×10^−4^ substitutions/site/year (**Table 8**). Therefore, the Spanish epidemic appears to be the most recent ongoing epidemic, followed by China and then Iran. The effective number of infections in Spain is higher compared with that in China, but the epidemic growth rate is slightly slower than in China. Likewise, the rate of molecular evolution of the Spanish epidemic is also the lowest among all three epidemics.

**Table 7:** Relevant estimates from the Bayesian analysis for the Iranian AMV population.

	Mean	SEM	Median	Lower 95% HPD	Higher 95% HPD
TMRCA	1996.2799	1.3588	1998.3026	2006.4692	1980.3316
Exponential population size	7.41E+06	4.56E+06	1.78E+05	1.50E+03	1.72E+07
Exponential growth rate	0.6687	0.043	0.6177	0.2563	1.1512
Mean rate of evolution	8.05E-04	4.95E-05	7.97E-04	2.88E-04	1.30E-03

**Table 8:** Relevant estimates from the Bayesian analysis for the Spanish AMV population.

	Mean	SEM	Median	Lower 95% HPD	Higher 95% HPD
TMRCA	1891.0391	5.4664	1907.1267	1970.3314	1772.6213
Exponential population size	1016.9043	73.0583	743.9589	165.36	2565.0284
Exponential growth rate	0.0431	2.10E-03	0.038	6.84E-03	0.0911
Mean rate of evolution	2.79E-04	8.22E-06	2.76E-04	9.79E-05	4.62E-04

BaTS found evidence of population structure at the geographic level in both Spain and Iran (Iran: *AI* = 7.192, *P* = 0.04, *PS* = 47.730, *P* = 0.04; Spain: *MC* = 1.9711, *P* = 0.014) (**Table 9**). No evidence of association of isolates by host species or families was found in either case. This again suggests a very recent introduction of the virus and a rapid spread among susceptible hosts without time to adapt to each possible host. For the Iranian population, no differences were detected by AMOVA among provinces or among and within localities (**Supplementary Table S9**). For the Spanish population, BaTS revealed a significant association between MCC tree clusters and geographic origin (*AI* = 2.366, *P* = 0.005), which was mostly driven by isolates from Zaragoza province (*MC* = 11.7938, *P* = 0.004) forming their own clusters (**Table 10**). This maybe indicate the idea that Zaragoza province is the geographic origin of the epidemic. BaTS did not find an association between the MCC tree clusters and the host species or families.

**Table 9:** BaTS algorithm analysis for the Iranian AMV population (provinces).

	Mean	SEM	Median	Lower 95% HPD	Higher 95% HPD	*p*
AI	7.19221			6.40572	7.96465	0.04
PS	47.72992			46	49	0.04
MC (Ardebil)						
MC (Azerbaijan)						
MC (Chahar Mahaal)						
MC (Es)	1.97111	2	2			0.014
MC (Fars)						
MC (Ga)						
MC (Golestan)						
MC (Hamadan)						
MC (Hormozgan)						
MC (Isfahan)						
MC (Kerman)						
MC (Khorasan)						
MC (Sistan)						
MC (Tehran)						
MC (Yazd)						
MC (Zanjan)						

**Table 10:** BaTS algorithm analysis for the Spanish AMV population (provinces).

	Mean	SEM	Median	Lower 95% HPD	Higher 95% HPD	*p*
AI	2.36567			1.76494	2.96247	0.005
PS	15.68292			14	17	0.05
MC (Almeria)						
MC (Badajoz)						
MC (Girona)						
MC (Huesca)						
MC (Leon)						
MC (Malaga)						
MC (Zaragoza)	11.7938			9	15	0.004

#### 3.3.2 Selection analyses

Selection analysis of the Iranian population shows several cases of negative selection and a distinct case of positive selection in **Figure 2B** and **Supplementary Table S10**. For the Spanish population, the selection is predominantly negative (**Figure 2C** and **Supplementary Table S11**). Furthermore, using AMOVA, a significant difference was found within Spanish provinces **(** *P* = 0.0026**)** (**Supplementary Table S12**) and potentially a few sites with weakly positive selection, but not the same as that suggested for China and Iran.

#### 3.3.3 Population structure

In the Iranian population, the very rapid spread of the epidemic has not allowed the establishment of significant population differentiation among geographic locations (**Table 1**); essentially it is a single pandemic population. In the Spanish population, significant differences only exist between samples taken from the same geographic location and not among locations from the same province (likewise the case in China) (**Tables 2**, **3**). Moreover, based on the host at species and family levels, the Spanish population exhibits significant differences among the hosts (**Supplementary Tables S13**, **S14** **),** while the Chinese population only shows a significant difference at the host family level (**Tables 4**, **5**); for the Iranian population, there was no differences among the hosts at species or family levels (**Supplementary Tables S15**, **S16**).

## 4 Discussion

This study conducted an in-depth and comprehensive analysis of AMV populations from China (86), Iran (91), and Spain (56) using the whole *cp* gene sequence. In the research of Bergua et al. (2014), only 390 nt of this gene sequence was used for the phylogenetic analysis, resulting in the loss of some evolution information of the *cp* gene. In the work of Xu and Nie (2006), only eight isolates were analyzed phylogenetically, which also limited the research and conclusions. Our study is the first to explore the evolution of the AMV population from Iran. Comparing the patterns across the three different AMV epidemics, it could be concluded Iran has the longest epidemic history and this is still rapidly expanding, while the epidemics of China and Spain are younger. This is the first comparison of the AMV populations of these three countries.

Both Mushegian and Morris and their respective colleagues stated that all viral contributions to plant ecosystem function must derive from the complex interactions between viruses, plants, and transmission vectors (Mushegian et al., 2016; Morris et al., 2018), thus, the plant host and transmission vectors will also affect the evolution of the viruses. Viruses need to rapidly adapt to changes in host genotypes (Stroud and Losos, 2016). On the one hand, specialist viruses may undergo adaptive radiation when they are in heterogeneous habitats, resulting in increased diversity of the population (Lefeuvre et al., 2019). This is the case for the Spanish population at the host species and family levels and the Chinese population at the host family level, where significant differences were detected. On the other hand, generalist viruses that infect multiple host species for survival compete with other viruses for the resources (Scholthof et al., 2011; Jacquemond, 2012). Such a situation should yield a low-diversity viral population dominated by one or a few of the best-adapted viral genotypes (Stroud and Losos, 2016), as seen with the Chinese AMV population at the host species level and the Iranian population at the host species and family levels. From the host species of the AMV in Spain have much more than China, which reflected the diversity of broad-spectrum of hosts of AMV. As the fastest evolving population, the Iranian AMV population has the highest exponential population size and exponential growth rate for the extensive adaptability to hosts. Moreover, as a specialist or as a generalist, the virus also depends on the feeding preferences of its vectors (Bragard et al., 2013; Dietzgen et al., 2016). From the significant differences among the hosts at the family and species levels, the diversity of the population also reflects the diversities of the transmission vectors while simultaneously reflecting complex geographical environments and the diversity of crops suitable for planting (Lane and Jarvis, 2007; Simmonds et al., 2019).

A vast territory naturally increases the diversity of the geographical environment, plants, and transmission vectors, and directly leads to the highest evolution rate of viruses to adaptation. In the AMV population of China, there were significant differences in AMV isolates among the provinces and bioclimatic zones. Large differences in topography, landforms, longitude, and latitude among various provinces results in some differences in the microenvironment for the plants (Wang et al., 2015), and AMV needs to adapt to these. Moreover, in the subtropical monsoon climate zone, a significant difference existed in the population, which consisted of isolates from 10 locations; the western-most location is Dali (E100.31 N25.63), the eastern-most location is Hangzhou (E120.20 N30.23), and the distance between the two sites is approximately 2016.8 kilometers. Collectively, these factors resulted in the Chinese population having the fastest rate of molecular evolution.

A significant difference was observed among different locations within the same province for the Chinese and Spanish populations. The geographic distances among locations might explain this phenomenon; moreover, agricultural practices may also be one of the influencing factors. In the past 50 years, the development of agricultural production, frequent introductions of new cultivars, and the changing cropping system may have posed strong selective pressures upon AMV, resulting in rapid evolution. Although there was no difference in isolates among provinces within the same climatic zone, significant differences between isolates at different sampling points in the same province were found. This is likely due to differences in farming systems, which should be the collateral impacts of human activities on these ecological roles and bring a particularly powerful and quantitative effect on plant virus evolution (Lefeuvre et al., 2019).

Within the Chinese population, whether based on the host diversity of isolates or the variability of geographical location and climatic zone, a very rich diversity was observed. The Iranian population, representing the earliest introduction of AMV in Asia and showing the fastest evolution among the three countries analyzed, showed no significant difference among the host plant species. The Spanish population, with the origin of AMV found in Zaragoza, exhibited the slowest spread and the slowest evolution rate among the three countries. The phylogenetic tree was not divided into different populations according to the host species and most of the evolutionary selection was negative selection, with a few sites showing positive selection, which was different from the Chinese and Iranian populations. The significant differences among different isolates in the same location and the significant differences among different sampling sites in the same province were similar to those of the Chinese population.

For the Chinese population, there are questions as to whether AMV evolution is a Russian doll model and whether AMV epidemiology is “fractal”. Continents are homogenous while countries are heterogeneous, so do provinces within countries, while locations within province. If the unit of research is set at the country level, the provinces within the country are homogeneous, locations within provinces are homogeneous (depending on the country—they are homogenous for Iran but not for China and Spain), and plants from the same location are heterogeneous (but not for Iran). In addition, there is a further question as to why the AMV populations from China and Spain have the most genetic diversity the lower you go on the geographic scale? With the development of the economy of the world, the economic activities, such as the commercial activities in agriculture, especially the import and export transactions of various countries for alfalfa quality, are important factors affecting the distribution and diversity of AMV isolates. For example, with the development of China’s economy and the improvement in living standards, a large number of imports of alfalfa are needed in China to meet domestic demand; consequently, foreign AMV isolates have entered China from Canada, USA, and Spain, etc. (Yuan et al., 2015). The differences between the isolates in Hangzhou of Zhejiang Province and Cangzhou of Hebei Province, respectively, are significant, and the main reason for this is likely related to the exchange between alfalfa varieties of China and foreign countries.

In summary, AMV populations in China, Iran, and Spain show rich diversity through evolution, and owing to the participation of human activities, the population evolution of AMV will gradually present more new characteristics. This warrants the need for continued research on AMV to facilitate the detection and early warning of plant virus diseases.

## Data availability statement

The datasets presented in this study can be found in online repositories. The names of the repository/repositories and accession number(s) can be found in the article/**Supplementary Material**.

## Author contributions

XW, CL and ZT performed experiments. JZ and RW isolated some isolates. YW and XJ conceptualized experiments. BW designed the study, analyzed data, and wrote the manuscript. All authors contributed to the article and approved the submitted version.
