# Supplementary material for: Population genetics and phylogeography of alfalfa mosaic virus in China and a comparison with other regional epidemics based on the cp gene
Source: Front Plant Sci. 2023 Feb 14;13:1105198. doi: 10.3389/fpls.2022.1105198 (PMC9971725; doi:10.3389/fpls.2022.1105198)
Supplement: Supplementary file 1 [file DataSheet_1.docx]

Supplementary Material

**Population genetics and phylogeography of alfalfa mosaic virus in China and a comparison with other regional epidemics based on the *cp* gene**

**Xin Wang^1,2^**^†^**, Chenchen Liu^1^**^†^**, Zhaoyan Tan^1^**^†^**, Jiantai Zhang^1,2^, Rongqun Wang^1^, Yuanhong Wang^3^, Xiliang Jiang^1^, Beilei Wu^1^***

First Author^†^: Co-Author

*** Correspondence:** Corresponding Author: [blwu@ippcaas.cn](mailto:blwu@ippcaas.cn)

# Supplementary Figures

**
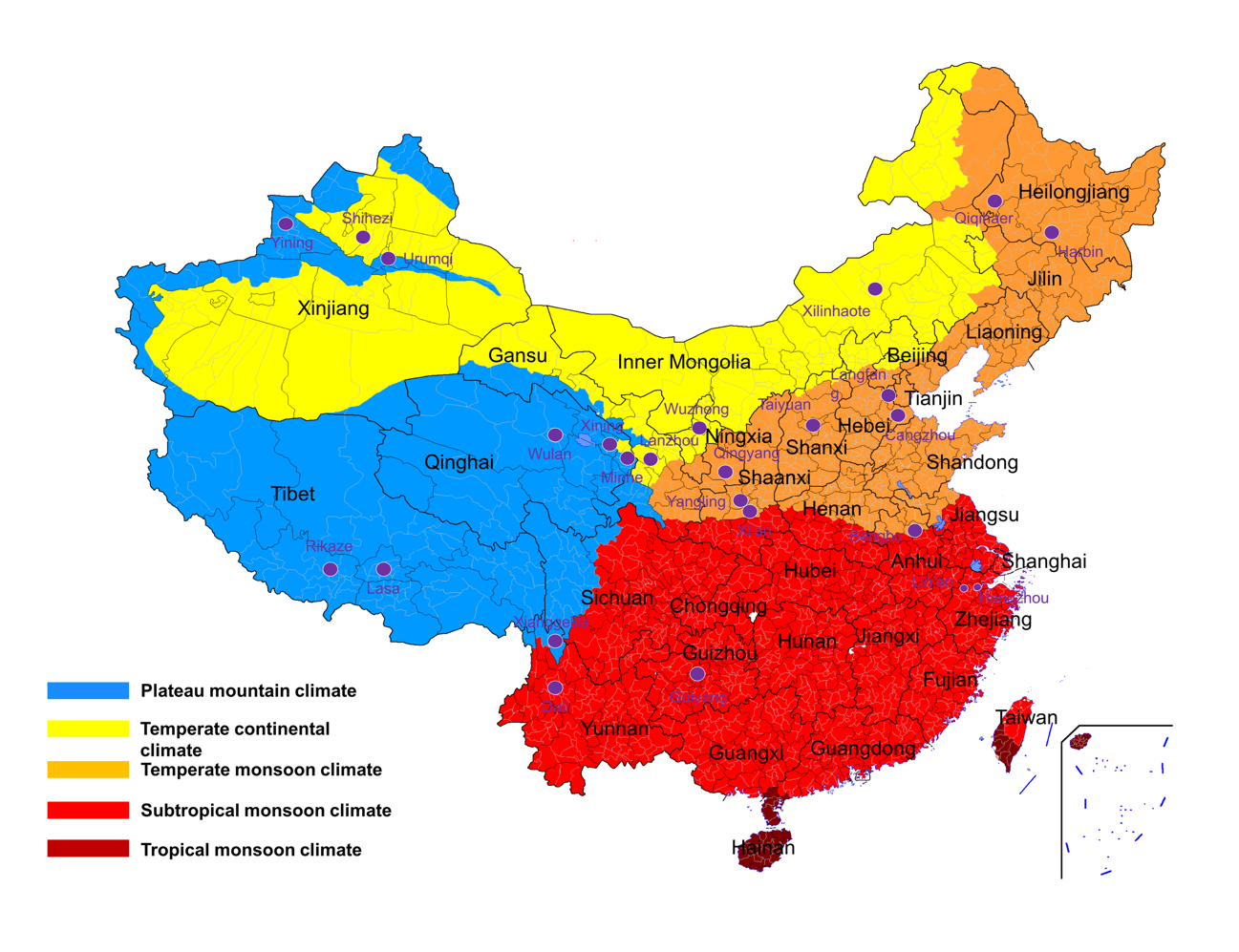
**

**Supplementary Figure S1.** Locations of isolates in the climatic zones of China.


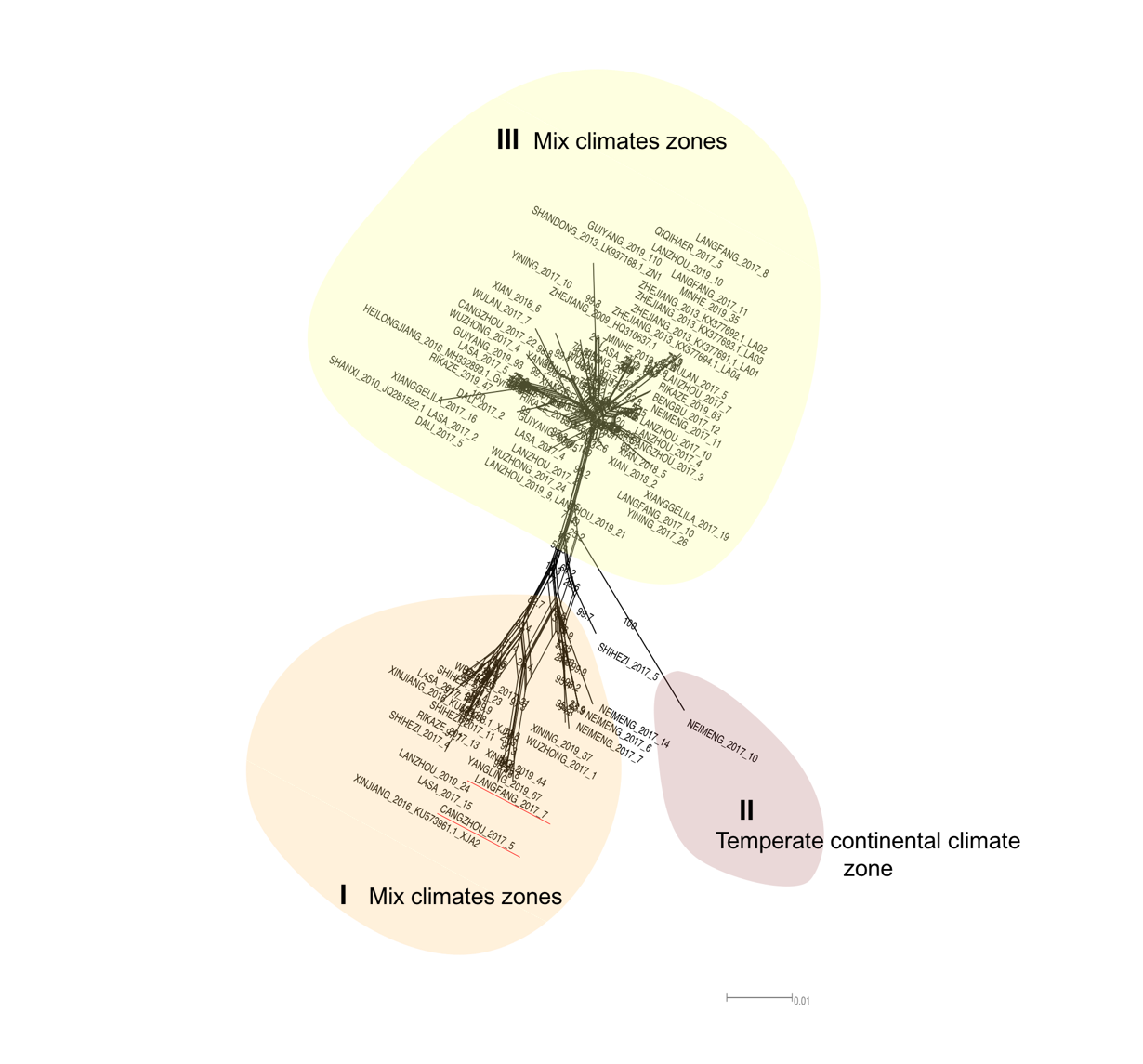


**Supplementary Figure S2.** Parsimony networks of AMV population from China


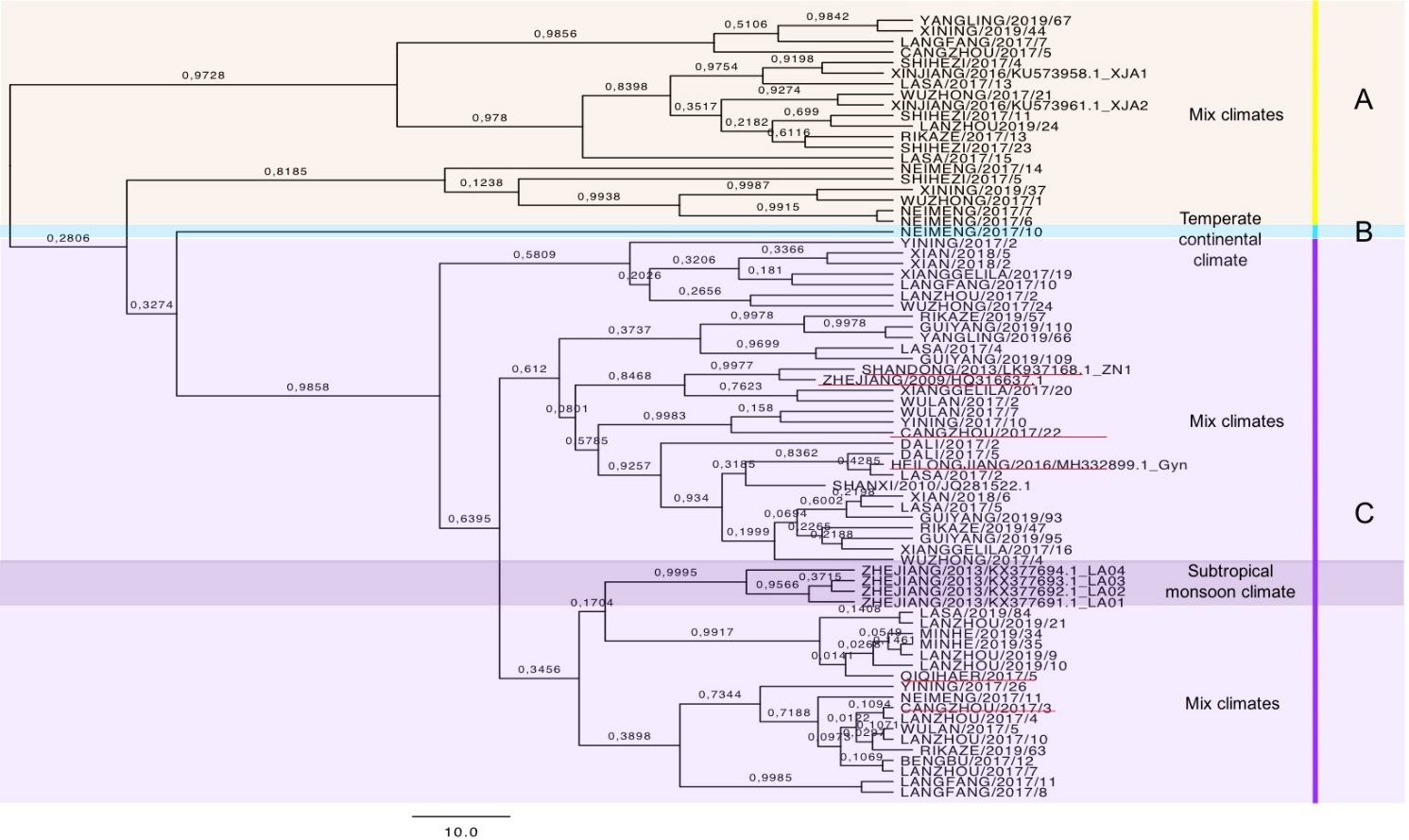


**Supplementary Figure S3.** MCC tree obtained with BEAST for the AMV population from China.


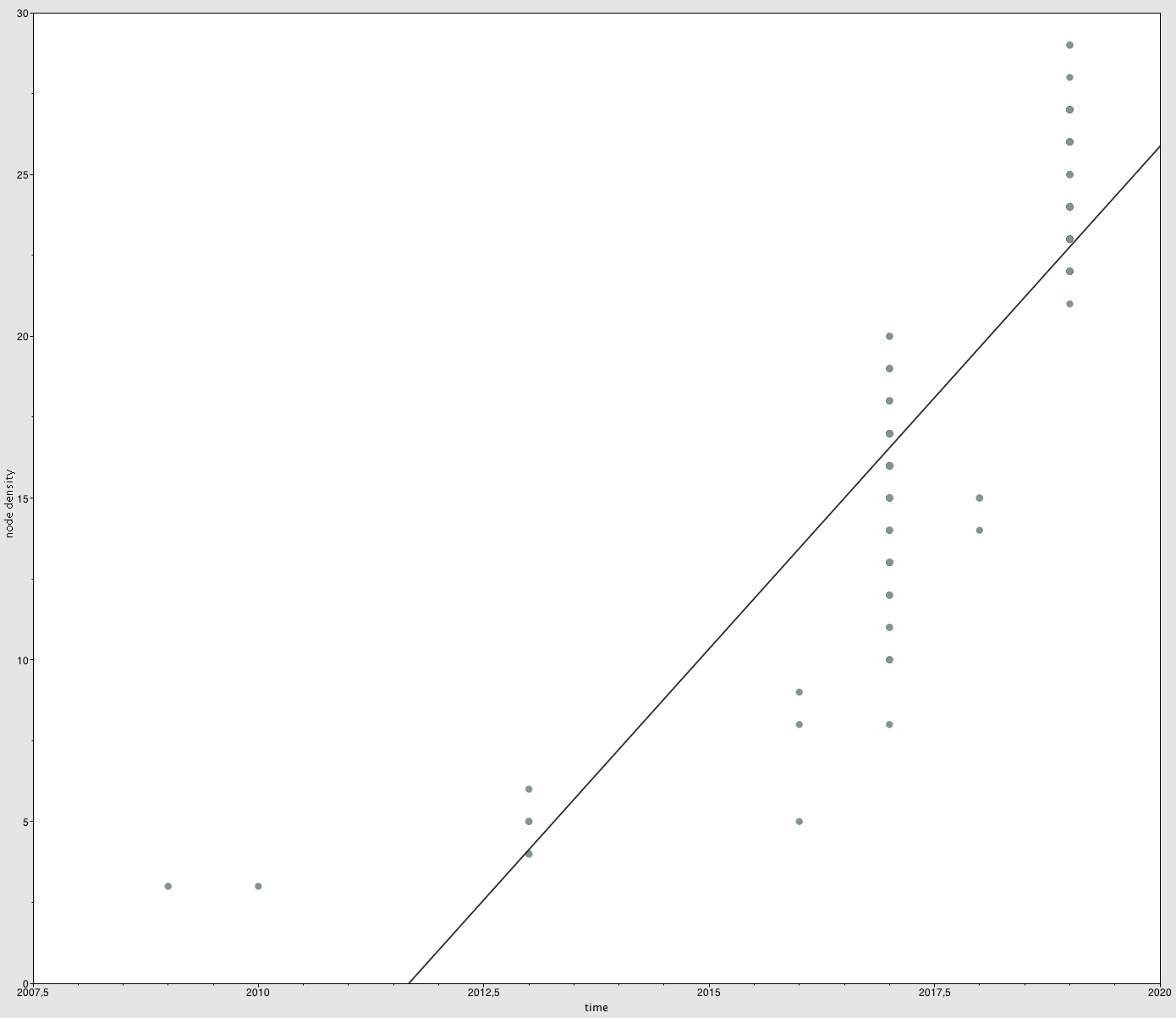


**Supplementary Figure S4.** TempEst found a significant time-stamp in the amount of divergence in the Chinese AMV population

**Supplementary Figure S5.** Parsimony network MCC tree of isolates from Iran

**Supplementary Figure S6.** Parsimony network MCC tree of isolates from Spain

**Supplementary Figure S7.** MCC tree obtained with BEAST for the AMV population from Spain.


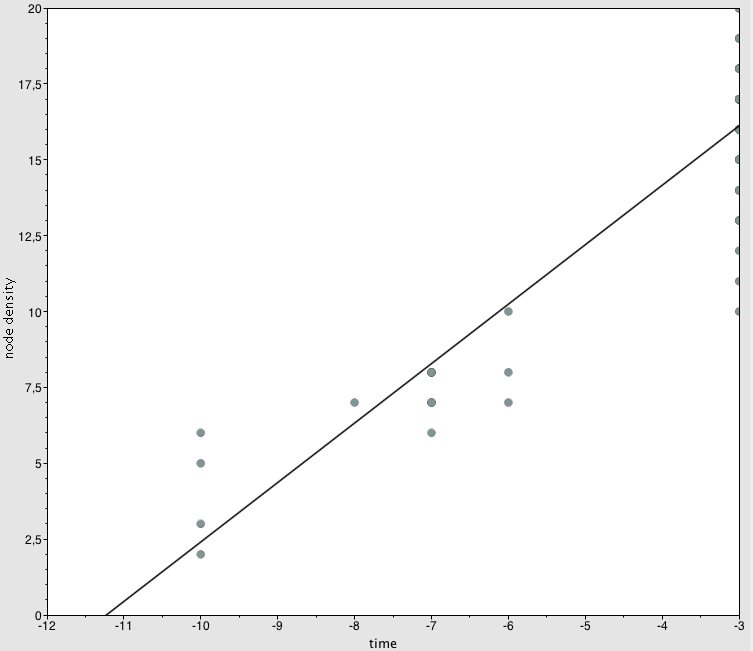


**Supplementary Figure S8**. TempEst found a significant time-stamp in the amount of divergence in the Iranian AMV population


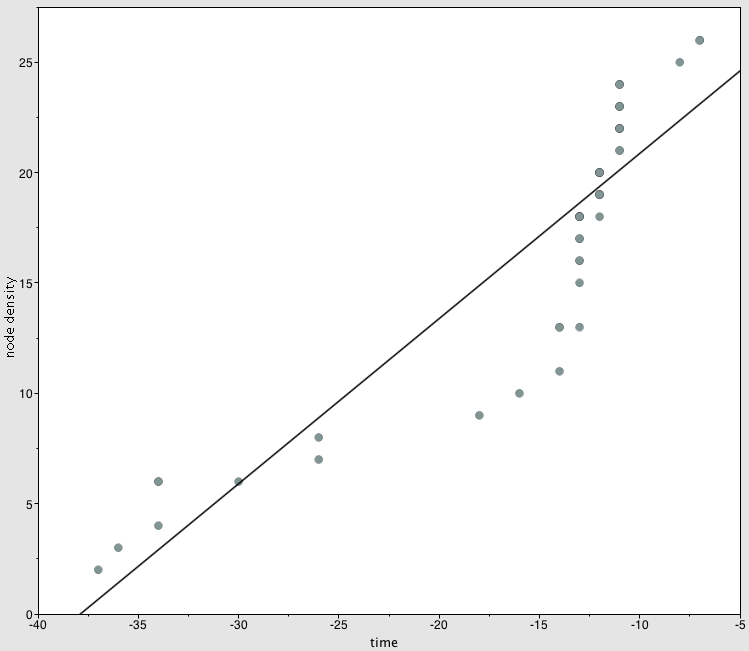


**Supplementary Figure S9.** TempEst found a significant time-stamp in the amount of divergence in the Spanish AMV population

## Supplementary Tables

**Supplementary Table S1.** Alfalfa mosaic virus isolates—including host, collection date, and location—obtained from China in this study.

| **Accession number** | **The *cp* gene from isolates of Alfalfa mosaic virus from China** | **Country** | **Collection date** | **Host** | **Longitude and latitude** |
| --- | --- | --- | --- | --- | --- |
| MW633762 | YINING-CP/2017/26 | Yining, Xinjiang | 2-Oct-17 | *Medicago sativa* | 44.52°N, 81.55°E |
| MW633763 | YINING-CP /2017/10 | Yining, Xinjiang | 2-Oct-17 | *Medicago sativa* | 44.52°N, 81.55°E |
| MW633764 | YINING-CP /2017/2 | Yining, Xinjiang | 2-Oct-17 | *Medicago sativa* | 44.52°N, 81.55°E |
| MW633765 | XIANGGELILA-CP /2017/20 | Shangri-la,Yunnan | 19-Jul-17 | *Medicago sativa* | 27.53°N, 99.81 |
| MW633766 | XIANGGELILA-CP /2017/19 | Shangri-la,Yunnan | 19-Jul-17 | *Medicago sativa* | 27.53°N, 99.81 |
| MW633767 | XIANGGELILA-CP /2017/16 | Shangri-la,Yunnan | 19-Jul-17 | *Medicago sativa* | 27.53°N, 99.81 |
| MW633768 | XIAN-CP /2018/6 | Xi'an, Shanxi | 17-Apr-18 | *Medicago sativa* | 16.56°N, 108.9°E |
| MW633769 | XIAN-CP /2018/5 | Xi'an, Shanxi | 17-Apr-18 | *Medicago sativa* | 16.56°N, 108.9°E |
| MW633770 | XIAN-CP /2018/2 | Xi'an, Shanxi | 17-Apr-18 | *Medicago sativa* | 16.56°N, 108.9°E |
| MW633771 | WUZHONG-CP /2017/24 | Ningxia,Wuzhong | 18-Aug-17 | *Medicago sativa* | 37.41°N, 106.53°E |
| MW633772 | WUZHONG-CP /2017/21 | Ningxia,Wuzhong | 18-Aug-17 | *Medicago sativa* | 37.41°N, 106.53°E |
| MW633773 | WUZHONG-CP /2017/4 | Ningxia,Wuzhong | 18-Aug-17 | *Medicago sativa* | 37.41°N, 106.53°E |
| MW633774 | WUZHONG-CP /2017/1 | Ningxia,Wuzhong | 18-Aug-17 | *Medicago sativa* | 37.41°N, 106.53°E |
| MW633775 | WULAN-CP /2017/7 | Ulan, Qinghai | 18-Aug-17 | *Medicago sativa* | 36.66°N, 99.29°E |
| MW633776 | WULAN-CP /2017/5 | Ulan, Qinghai | 18-Aug-17 | *Medicago sativa* | 36.66°N, 99.29°E |
| MW633777 | WULAN-CP /2017/2 | Ulan, Qinghai | 18-Aug-17 | *Medicago sativa* | 36.66°N, 99.29°E |
| MW633778 | SHIHEZI-CP /2017/23 | Shihezi, Xinjiang | 27-Sep-17 | *Medicago sativa* | 44.45°N, 85.56°E |
| MW633779 | SHIHEZI-CP /2017/11 | Shihezi, Xinjiang | 27-Sep-17 | *Medicago sativa* | 44.45°N, 85.56°E |
| MW633780 | SHIHEZI-CP /2017/5 | Shihezi, Xinjiang | 27-Sep-17 | *Medicago sativa* | 44.45°N, 85.56°E |
| MW633781 | SHIHEZI-CP /2017/4 | Shihezi, Xinjiang | 27-Sep-17 | *Medicago sativa* | 44.45°N, 85.56°E |
| MW633782 | RIKAZE-CP /2017/13 | Shigatse, Tibet | 10-Aug-17 | *Medicago sativa* | 29.45°N, 89.46°E |
| MW633783 | QIQIHAER-CP /2017/5 | Qiqihar,Heilongjiang | 12-Jun-17 | *Medicago sativa* | 47.35°N, 123.9°E |
| MW633784 | NEIMENG-CP /2017/14 | Xilin Hot, Neimeng | 1-Sep-17 | *Medicago sativa* | 43.04°N, 115.31°E |
| MW633785 | NEIMENG-CP /2017/11 | Xilin Hot, Neimeng | 1-Sep-17 | *Medicago sativa* | 43.04°N, 115.31°E |
| MW633786 | NEIMENG-CP /2017/10 | Xilin Hot, Neimeng | 1-Sep-17 | *Medicago sativa* | 43.04°N, 115.31°E |
| MW633787 | NEIMENG-CP /2017/7 | Xilin Hot, Neimeng | 1-Sep-17 | *Medicago sativa* | 43.04°N, 115.31°E |
| MW633788 | NEIMENG-CP /2017/6 | Xilin Hot, Neimeng | 1-Sep-17 | *Medicago sativa* | 43.04°N, 115.31°E |
| MW633789 | LASA-CP /2017/15 | Lhasa, Tibet | 11-Aug-17 | *Medicago sativa* | 29.6°N, 91.1°E |
| MW633790 | LASA-CP /2017/13 | Lhasa, Tibet | 11-Aug-17 | *Medicago sativa* | 29.6°N, 91.1°E |
| MW633791 | LASA-CP /2017/5 | Lhasa, Tibet | 11-Aug-17 | *Medicago sativa* | 29.6°N, 91.1°E |
| MW633792 | LASA-CP /2017/4 | Lhasa, Tibet | 11-Aug-17 | *Medicago sativa* | 29.6°N, 91.1°E |
| MW633793 | LASA-CP /2017/2 | Lhasa, Tibet | 11-Aug-17 | *Medicago sativa* | 29.6°N, 91.1°E |
| MW633794 | LASA-CP /2019/1 | Lhasa, Tibet | 11-Aug-17 | *Medicago sativa* | 29.6°N, 91.1°E |
| MW633795 | LASA-CP /2019/4 | Lhasa, Tibet | 11-Aug-17 | *Medicago sativa* | 29.6°N, 91.1°E |
| MW633796 | LASA-CP /2019/5 | Lhasa, Tibet | 11-Aug-17 | *Medicago sativa* | 29.6°N, 91.1°E |
| MW633797 | LASA-CP /2019/8 | Lhasa, Tibet | 11-Aug-17 | *Medicago sativa* | 29.6°N, 91.1°E |
| MW633798 | LASA-CP /2019/12 | Lhasa, Tibet | 11-Aug-17 | *Medicago sativa* | 29.6°N, 91.1°E |
| MW633799 | LANZHOU-CP /2017/10 | Lanzhou, Gansu | 15-Aug-17 | *Medicago sativa* | 36.05°N, 103.83°E |
| MW633800 | LANZHOU-CP /2017/7 | Lanzhou, Gansu | 15-Aug-17 | *Medicago sativa* | 36.05°N, 103.83°E |
| MW633801 | LANZHOU-CP /2017/4 | Lanzhou, Gansu | 15-Aug-17 | *Medicago sativa* | 36.05°N, 103.83°E |
| MW633802 | LANZHOU-CP /2017/2 | Lanzhou, Gansu | 15-Aug-17 | *Medicago sativa* | 36.05°N, 103.83°E |
| MW633803 | LANZHOU-CP /2019/24 | Lanzhou, Gansu | 15-Aug-17 | *Medicago sativa* | 36.05°N, 103.83°E |
| MW633804 | LANZHOU-CP /2019/21 | Lanzhou, Gansu | 15-Aug-17 | *Medicago sativa* | 36.05°N, 103.83°E |
| MW633805 | LANZHOU-CP /2019/10 | Lanzhou, Gansu | 15-Aug-17 | *Medicago sativa* | 36.05°N, 103.83°E |
| MW633806 | LANZHOU-CP /2019/9 | Lanzhou, Gansu | 15-Aug-17 | *Medicago sativa* | 36.05°N, 103.83°E |
| MW633807 | LANGFANG-CP /2017/11 | Langfang,Hebei | 8-Jun-17 | *Medicago sativa* | 44.1°N, 116.63°E |
| MW633808 | LANGFANG-CP /2017/10 | Langfang,Hebei | 8-Jun-17 | *Medicago sativa* | 44.1°N, 116.63°E |
| MW633809 | LANGFANG-CP /2017/8 | Langfang,Hebei | 8-Jun-17 | *Medicago sativa* | 44.1°N, 116.63°E |
| MW633810 | LANGFANG-CP /2017/7 | Langfang,Hebei | 8-Jun-17 | *Medicago sativa* | 44.1°N, 116.63°E |
| MW633811 | DALI-CP /2017/5 | Dali,Yunnan | 18-Jul-17 | *Medicago sativa* | 26.14°N, 100.02°E |
| MW633812 | DALI-CP /2017/2 | Dali,Yunnan | 18-Jul-17 | *Medicago sativa* | 26.14°N, 100.02°E |
| MW633813 | CANGZHOU-CP /2017/22 | Cangchou,Hebei | 21-Jun-17 | *Medicago sativa* | 38.5°N, 117.38°E |
| MW633814 | CANGZHOU-CP /2017/5 | Cangchou,Hebei | 21-Jun-17 | *Medicago sativa* | 38.5°N, 117.38°E |
| MW633815 | CANGZHOU-CP /2017/3 | Cangchou,Hebei | 21-Jun-17 | *Medicago sativa* | 38.5°N, 117.38°E |
| MW633816 | BENGBU-CP /2017/12 | Bengbu, Anhui | 21-Jun-17 | *Medicago sativa* | 32.95°N, 117.2°E |
| MW633817 | YANGLING-CP /2019/3 | Yangling,Shanxi | 21-Sep-19 | *Medicago sativa* | 34.16°N, 108.4°E |
| MW633818 | YANGLING-CP /2019/4 | Yangling,Shanxi | 21-Sep-19 | *Medicago sativa* | 34.16°N, 108.4°E |
| MW633819 | GUIYANG-CP /2019/8 | Guiyang,Guizhou | 23-Sep-19 | *Medicago sativa* | 26.35°N, 106.42°E |
| MW633820 | GUIYANG-CP /2019/2 | Guiyang,Guizhou | 23-Sep-19 | *Medicago sativa* | 26.35°N, 106.42°E |
| MW633821 | GUIYANG-CP /2019/4 | Guiyang,Guizhou | 23-Sep-19 | *Medicago sativa* | 26.35°N, 106.42°E |
| MW633822 | GUIYANG-CP /2019/18 | Guiyang,Guizhou | 23-Sep-19 | *Medicago sativa* | 26.35°N, 106.42°E |
| MW633823 | GUIYANG-CP /2019/19 | Guiyang,Guizhou | 23-Sep-19 | *Medicago sativa* | 26.35°N, 106.42°E |
| MW633824 | XINING-CP /2019/2 | Xining,Qinghai | 18-Sep-19 | *Medicago sativa* | 36.62°N, 101.77°E |
| MW633825 | XINING-CP /2019/6 | Xining,Qinghai | 18-Sep-19 | *Medicago sativa* | 36.62°N, 101.77°E |
| MW633826 | XINING-CP /2019/1 | Xining,Qinghai | 18-Sep-19 | *Medicago sativa* | 36.62°N, 101.77°E |
| MW633827 | XINING-CP /2019/8 | Xining,Qinghai | 18-Sep-19 | *Medicago sativa* | 36.62°N, 101.77°E |
| MW633828 | MINHE-CP /2019/6 | Minhe,Qinghai | 17-Sep-19 | *Medicago sativa* | 36.3°N, 102.58°E |
| MW633829 | MINHE-CP /2019/1 | Minhe,Qinghai | 17-Sep-19 | *Medicago sativa* | 36.3°N, 102.58°E |
| MW633830 | MINHE-CP /2019/7 | Minhe,Qinghai | 17-Sep-19 | *Medicago sativa* | 36.3°N, 102.58°E |
| MW633831 | YANGLING-CP /2019/9 | Yangling,Shanxi | 21-Sep-19 | *Medicago sativa* | 34.16°N, 108.4°E |
| MW633832 | YANGLING-CP /2019/12 | Yangling,Shanxi | 21-Sep-19 | *Medicago sativa* | 34.16°N, 108.4°E |
| MW633833 | RIKAZE-CP /2019/5 | Shigatse, Tibet | 12-Sep-19 | *Medicago sativa* | 29.45°N, 89.46°E |
| MW633834 | RIKAZE-CP /2019/8 | Shigatse, Tibet | 12-Sep-19 | *Medicago sativa* | 29.45°N, 89.46°E |
| MW633835 | RIKAZE-CP /2019/2 | Shigatse, Tibet | 12-Sep-19 | *Medicago sativa* | 29.45°N, 89.46°E |
| MW633836 | RIKAZE-CP /2019/12 | Shigatse, Tibet | 12-Sep-19 | *Medicago sativa* | 29.45°N, 89.46°E |
| MW633837 | RIKAZE-CP /2019/18 | Shigatse, Tibet | 12-Sep-19 | *Medicago sativa* | 29.45°N, 89.46°E |

**Supplementary Table S2.** Alfalfa mosaic virus isolates—including host, collection date, and location—obtained from the GenBank database.

| **GenBank accession number** | **Country** | **Collection date** | **Host** | **Isolate** | **Longitude and latitude** |
| --- | --- | --- | --- | --- | --- |
| LK937168 | Qingyang, Gansu, China | 2013 | *Nicotiana tabacum* cv. Qinyan96 | ZN-1 | 36.05°N, 108.46°E |
| JQ281522 | Taiyuan, Shanxi, China | 2010 |  |  | 39.9°N, 116.4°E |
| KX377694 | Lin'an, Zhejiang, China | 2013 | *Trifolium pratense* |  | 30.38°N, 120.2°E |
| KX377693 | Lin'an, Zhejiang, China | 2013 | *Veronica persica* Poir |  | 30.38°N, 120.2°E |
| KX377692 | Lin'an, Zhejiang, China | 2013 | *Justicia procumbens* |  | 30.38°N, 120.2°E |
| KX377691 | Lin'an, Zhejiang, China | 2013 | *Cayratia japonica* (Thunb.) Gagnep. |  | 30.38°N, 120.2°E |
| KU573961 | Urumqi, Xinjiang, China | 31-Jul-16 | *Medicago sativa* | XJA-2 | 39.9°N, 116.4°E |
| KU573958 | Urumqi, Xinjiang, China | 31-Jul-16 | *Medicago sativa* | XJA-1 | 39.9°N, 116.4°E |
| HQ316637.1 | Hangzhou, Zhejiang, China | Oct-09 | *Nicotiana glutinosa* |  | 39.9°N, 116.4°E |
| MH332899.1 | Harbin, Heilongjiang, China | 2016 | *Gynostemma pentaphyllum* | AMV-Gyn | 39.9°N, 116.4°E |
| KX535525 | Iran | 31-Jan-15 | *Plantago* sp. | Kh.Sa.Pl | 35.73°N, 51.5°E |
| KX535524 | Iran | 31-Jan-15 | *Rumex* sp. | Ke.Si.Ru | 35.73°N, 51.5°E |
| KX535523 | Iran | 31-Jan-15 | *Ammi majus* | Ke.Or.Am | 35.73°N, 51.5°E |
| KX535522 | Iran | 31-Jan-15 | *Trogopogon* sp. | Ke.Sh.Tr | 35.73°N, 51.5°E |
| KX535521 | Iran | 31-Jan-15 | *Trogopogon* sp. | Ho.Ba.Tr | 35.73°N, 51.5°E |
| KX535520 | Iran | 31-Jan-15 | *Sonchus asper* | Kh.Sa.So | 35.73°N, 51.5°E |
| KX535519 | Iran | 31-Jan-15 | *Sonchus asper* | Ho.Ba.So | 35.73°N, 51.5°E |
| KX535518 | Iran | 31-Jan-15 | *Dacus carota* | Ch.Fa.Da | 35.73°N, 51.5°E |
| KX535517 | Iran | 31-Jan-15 | *Chenopodium album* | Ke.Sh.Ch | 35.73°N, 51.5°E |
| KX535516 | Iran | 31-Jan-15 | *Chenopodium album* | Ke.Sa.Ch | 35.73°N, 51.5°E |
| KX535515 | Iran | 31-Jan-15 | *Chenopodium album* | Es.Jo.Ch | 35.73°N, 51.5°E |
| KX535514 | Iran | 31-Jan-15 | *Capsicum annuum* | Ke.Sa.Pe | 35.73°N, 51.5°E |
| KX535513 | Iran | 31-Jan-15 | *Capsicum annuum* | Ke.Ke.Pe | 35.73°N, 51.5°E |
| KX535512 | Iran | 31-Jan-15 | *Solanum tuberosum* | Ke.Ji.Po | 35.73°N, 51.5°E |
| KX535511 | Iran | 31-Jan-15 | *Solanum tuberosum* | Ke.Ma.Po2 | 35.73°N, 51.5°E |
| KX535510 | Iran | 31-Jan-15 | *Solanum tuberosum* | Ke.Ma.Po | 35.73°N, 51.5°E |
| KX535509 | Iran | 31-Jan-15 | *Solanum tuberosum* | Ke.Ke.Po | 35.73°N, 51.5°E |
| KX535508 | Iran | 31-Jan-15 | *Solanum tuberosum* | Ha.Da.Po | 35.73°N, 51.5°E |
| KX535507 | Iran | 31-Jan-15 | *Solanum tuberosum* | Es.Fa.Po | 35.73°N, 51.5°E |
| KX535506 | Iran | 31-Jan-15 | *Medicago sativa* | Za.Es.A | 35.73°N, 51.5°E |
| KX535505 | Iran | 31-Jan-15 | *Medicago sativa* | Ya.Me.A | 35.73°N, 51.5°E |
| KX535504 | Iran | 31-Jan-15 | *Medicago sativa* | Ya.Ha.A | 35.73°N, 51.5°E |
| KX535503 | Iran | 31-Jan-15 | *Medicago sativa* | Ya.Za.A | 35.73°N, 51.5°E |
| KX535502 | Iran | 31-Jan-15 | *Medicago sativa* | Te.Ka.A | 35.73°N, 51.5°E |
| KX535501 | Iran | 31-Jan-15 | *Medicago sativa* | Si.Zb.A | 35.73°N, 51.5°E |
| KX535500 | Iran | 31-Jan-15 | *Medicago sativa* | Si.Ze.A | 35.73°N, 51.5°E |
| KX535499 | Iran | 31-Jan-15 | *Medicago sativa* | Si.Ni.A | 35.73°N, 51.5°E |
| KX535498 | Iran | 31-Jan-15 | *Medicago sativa* | Si.Za.A | 35.73°N, 51.5°E |
| KX535497 | Iran | 31-Jan-15 | *Medicago sativa* | Si.Za.A1 | 35.73°N, 51.5°E |
| KX535496 | Iran | 31-Jan-15 | *Medicago sativa* | Kh.Ma.A | 35.73°N, 51.5°E |
| KX535495 | Iran | 31-Jan-15 | *Medicago sativa* | Kh.Bj.A | 35.73°N, 51.5°E |
| KX535494 | Iran | 31-Jan-15 | *Medicago sativa* | Kh.Ah.A | 35.73°N, 51.5°E |
| KX535493 | Iran | 31-Jan-15 | *Medicago sativa* | Kh.Be.A3 | 35.73°N, 51.5°E |
| KX535492 | Iran | 31-Jan-15 | *Medicago sativa* | Kh.Be.A2 | 35.73°N, 51.5°E |
| KX535491 | Iran | 31-Jan-15 | *Medicago sativa* | Kh.Be.A1 | 35.73°N, 51.5°E |
| KX535490 | Iran | 31-Jan-15 | *Medicago sativa* | Kh.Be.A | 35.73°N, 51.5°E |
| KX535489 | Iran | 31-Jan-15 | *Medicago sativa* | Ke.Sa.A | 35.73°N, 51.5°E |
| KX535488 | Iran | 31-Jan-15 | *Medicago sativa* | Ke.Ma.A | 35.73°N, 51.5°E |
| KX535487 | Iran | 31-Jan-15 | *Medicago sativa* | Ho.Mi.A | 35.73°N, 51.5°E |
| KX535486 | Iran | 31-Jan-15 | *Medicago sativa* | Ha.Ba.A | 35.73°N, 51.5°E |
| KX535485 | Iran | 31-Jan-15 | *Medicago sativa* | Go.Mi.A | 35.73°N, 51.5°E |
| KX535484 | Iran | 31-Jan-15 | *Medicago sativa* | Go.Go.A | 35.73°N, 51.5°E |
| KX535483 | Iran | 31-Jan-15 | *Medicago sativa* | Ga.Ta.A | 35.73°N, 51.5°E |
| KX535482 | Iran | 31-Jan-15 | *Medicago sativa* | Fa.Es.A | 35.73°N, 51.5°E |
| KX535481 | Iran | 31-Jan-15 | *Medicago sativa* | Fa.Fa.A | 35.73°N, 51.5°E |
| KX535480 | Iran | 31-Jan-15 | *Medicago sativa* | Fa.Sa.A | 35.73°N, 51.5°E |
| KX535479 | Iran | 31-Jan-15 | *Medicago sativa* | Fa.Ma.A | 35.73°N, 51.5°E |
| KX535478 | Iran | 31-Jan-15 | *Medicago sativa* | Es.Es.A | 35.73°N, 51.5°E |
| KX535477 | Iran | 31-Jan-15 | *Medicago sativa* | Es.Fi.A | 35.73°N, 51.5°E |
| KX535476 | Iran | 31-Jan-15 | *Medicago sativa* | Es.Go.A | 35.73°N, 51.5°E |
| KX535475 | Iran | 31-Jan-15 | *Medicago sativa* | Ch.Sh.A | 35.73°N, 51.5°E |
| KX535474 | Iran | 31-Jan-15 | *Medicago sativa* | Ar.Sa.A | 35.73°N, 51.5°E |
| KX535473 | Iran | 31-Jan-15 | *Medicago sativa* | Az.Ta.A | 35.73°N, 51.5°E |
| KX535472 | Iran | 31-Jan-15 | *Medicago sativa* | Az.Mi.A | 35.73°N, 51.5°E |
| KX535471 | Iran | 31-Jan-15 | *Medicago sativa* | Az.Sa.A | 35.73°N, 51.5°E |
| KM655880 | Iran | 27-Jul-11 | *Capsicum annuum* voucher PPB4 | PPB4 | 35.73°N, 51.5°E |
| KM655879 | Iran | 14-Jul-11 | *Chenopodium album* voucher WS | WS | 35.73°N, 51.5°E |
| KM655878 | Iran | 13-Sep-11 | *Vigna unguiculata* voucher TBN | TBN | 35.73°N, 51.5°E |
| KM655877 | Iran | 30-Jul-12 | *Lycopersicon esculentum* voucher LPK | LPK | 35.73°N, 51.5°E |
| KM655876 | Iran | 12-Oct-10 | *Capsicum frutescens* voucher PKH | PKH | 35.73°N, 51.5°E |
| KM655875 | Iran | 2-Oct-11 | *Capsicum annuum* voucher PPF | PPF | 35.73°N, 51.5°E |
| KM655874 | Iran | 2011 | *Solanum nigrum* voucher PSH | PSH | 35.73°N, 51.5°E |
| KM655873 | Iran | 2011 | *Solanum nigrum* voucher SMZ | SMZ | 35.73°N, 51.5°E |
| KM655872 | Iran | 2011 | *Medicago sativa* voucher AK2 | AK2 | 35.73°N, 51.5°E |
| KM655871 | Iran | 2011 | *Medicago sativa* voucher ADA4 | ADA4 | 35.73°N, 51.5°E |
| JQ673587 | Iran | 2008 | *Medicago sativa* | Ke.Ba.Al | 35.73°N, 51.5°E |
| JQ685860 | Iran | 2008 | *Solanum tuberosum* | Ke.Ba.Po | 35.73°N, 51.5°E |
| JQ685859 | Iran | 2008 | *Medicago sativa* | Ke.Si.Al | 35.73°N, 51.5°E |
| JQ685858 | Iran | 2008 | *Medicago sativa* | Ke.Sh.Al | 35.73°N, 51.5°E |
| JX853611 | Tehran, Iran | 2012 | *Campsis radicans* | IRN-Tru | 35.7°N, 51.41°E |
| JX853610 | Bahar, Hamadan, Iran | 2011 | *Medicago sativa* | AP1 | 32.48°N, 51, 76°E |
| KX535470.1 | Iran | 31-Jan-15 | *Sonchus asper* | Kh.Sa.So | 35.73°N, 51.5°E |
| KX535469.1 | Iran | 31-Jan-15 | *Plantago* sp. | Kh.Sa.Pl | 35.73°N, 51.5°E |
| KX535468.1 | Iran | 31-Jan-15 | *Chenopodium album* | Es.Jo.Ch | 35.73°N, 51.5°E |
| KX535467.1 | Iran | 31-Jan-15 | *Medicago sativa* | Si.Ze.A | 35.73°N, 51.5°E |
| KX535466.1 | Iran | 31-Jan-15 | *Medicago sativa* | Kh.Be.A3 | 35.73°N, 51.5°E |
| KX535465.1 | Iran | 31-Jan-15 | *Medicago sativa* | Kh.Be.A2 | 35.73°N, 51.5°E |
| KX535464.1 | Iran | 31-Jan-15 | *Medicago sativa* | Kh.Be.A1 | 35.73°N, 51.5°E |
| KX535463.1 | Iran | 31-Jan-15 | *Medicago sativa* | Kh.Be.A | 35.73°N, 51.5°E |
| KX535462.1 | Iran | 31-Jan-15 | *Medicago sativa* | Ke.Ma.A | 35.73°N, 51.5°E |
| KX535461.1 | Iran | 31-Jan-15 | *Medicago sativa* | Ho.Mi.A | 35.73°N, 51.5°E |
| KX535460.1 | Iran | 31-Jan-15 | *Medicago sativa* | Go.Go.A | 35.73°N, 51.5°E |
| KX535459.1 | Iran | 31-Jan-15 | *Medicago sativa* | Fa.Fa.A | 35.73°N, 51.5°E |
| KX535458.1 | Iran | 31-Jan-15 | *Medicago sativa* | Fa.Es.A | 35.73°N, 51.5°E |
| KX535457.1 | Iran | 31-Jan-15 | *Medicago sativa* | Es.Go.A | 35.73°N, 51.5°E |
| KX535456.1 | Iran | 31-Jan-15 | *Medicago sativa* | Es.Fi.A | 35.73°N, 51.5°E |
| KX535455.1 | Iran | 31-Jan-15 | *Medicago sativa* | Az.Sa.A | 35.73°N, 51.5°E |
| KX535454.1 | Iran | 31-Jan-15 | *Medicago sativa* | Az.Mi.A | 35.73°N, 51.5°E |
| KX535453.1 | Iran | 31-Jan-15 | *Medicago sativa* | Ya.Za.A | 35.73°N, 51.5°E |
| KX535452.1 | Iran | 31-Jan-15 | *Medicago sativa* | Ya.Ma.A | 35.73°N, 51.5°E |
| KX535451.1 | Iran | 31-Jan-15 | *Medicago sativa* | Ya.Ha.A | 35.73°N, 51.5°E |
| EF427449 | Spain | 23-Jul-16 | *Viburnum* sp. | AMV-VIRB | 40.41°N, 3.75′W |
| JQ691234 | Zaragoza, Spain | 2006 | *Sonchus oleraceus* | S.o-2-06 | 41.63°N, 0.88°W |
| JQ691230 | Zaragoza, Spain | 2006 | *Trifolium repens* | T.r-1-06 | 41.63°N, 0.88°W |
| JQ691229 | Zaragoza, Spain | 2005 | *Chenopodium album* | C.a-4-05 | 41.63°N, 0.88°W |
| JQ691227 | Zaragoza, Spain | 2006 | *Solanum nigrum* | S.n-1-06 | 41.63°N, 0.88°W |
| JQ691223 | Zaragoza, Spain | 2006 | *Vicia faba* | Hb-1-06 | 41.63°N, 0.88°W |
| JQ691221 | Zaragoza, Spain | 2007 | *Solanum lycopersicum* | T-4-07 | 41.63°N, 0.88°W |
| JQ691220 | Zaragoza, Spain | 2007 | *Solanum lycopersicum* | T-3-07 | 41.63°N, 0.88°W |
| JQ691219 | Zaragoza, Spain | 2005 | *Solanum lycopersicum* | T-12-05 | 41.63°N, 0.88°W |
| JQ691218 | Zaragoza, Spain | 2005 | *Solanum lycopersicum* | T-9-05 | 41.63°N, 0.88°W |
| JQ691216 | Zaragoza, Spain | 2005 | *Solanum lycopersicum* | T-7-05 | 41.63°N, 0.88°W |
| JQ691215 | Zaragoza, Spain | 1992 | *Solanum lycopersicum* | T-6-92 | 41.63°N, 0.88°W |
| JQ691210 | Leon, Spain | 2004 | *Capsicum annuum* | P-15-04 | 21.01°N, 101.7°W |
| JQ691209 | Leon, Spain | 2004 | *Capsicum annuum* | P-14-04 | 21.01°N, 101.7°W |
| JQ691203 | Gerona, Spain | 2007 | *Medicago sativa* | Alf-20-07 | 41.15°N, 2.75°E |
| JQ691195 | Gerona, Spain | 2007 | *Medicago sativa* | Alf-3-07 | 41.15°N, 2.75°E |
| JQ691193 | Zaragoza, Spain | 2007 | *Medicago sativa* | Alf-1-07 | 41.63°N, 0.88°W |
| JQ691191 | Zaragoza, Spain | 2006 | *Medicago sativa* | Alf-1-06 | 41.63°N, 0.88°W |
| JQ691190 | Huesca, Spain | 2005 | *Medicago sativa* | Alf-41-05 | 42.13°N, 0.4°W |
| JQ691185 | Zaragoza, Spain | 2005 | *Medicago sativa* | Alf-15-05 | 41.63°N, 0.88°W |
| JQ691184 | Zaragoza, Spain | 2005 | *Medicago sativa* | Alf-14-05 | 41.63°N, 0.88°W |
| JQ691179 | Zaragoza, Spain | 2005 | *Medicago sativa* | Alf-1-05 | 41.63°N, 0.88°W |
| JQ691178 | Zaragoza, Spain | 2004 | *Medicago sativa* | Alf-3-04 | 41.63°N, 0.88°W |
| HE591387 | Caleta de Velez (Malaga), Spain | 2011 | *Hibiscus rosa-sinensis* L. | CV1 | 41.63°N, 0.88°W |
| HE591386 | Torre del Mar (Malaga), Spain | 2011 | host leaf | TM2 | 41.15°N, 2.75°E |
| JQ691236 | Zaragoza, Spain | 2006 | *Malva sylvestris* | M.s-3-06 | 41.63°N, 0.88°W |
| JQ691235 | Zaragoza, Spain | 2006 | *Picris echioides* | P.e-1-06 | 41.63°N, 0.88°W |
| JQ691233 | Zaragoza, Spain | 2006 | *Calystegia sepium* | C.s-1-06 | 41.63°N, 0.88°W |
| JQ691232 | Zaragoza, Spain | 2006 | *Trifolium repens* | T.r-4-06 | 41.63°N, 0.88°W |
| JQ691231 | Zaragoza, Spain | 2006 | *Trifolium repens* | T.r-3-06 | 41.63°N, 0.88°W |
| JQ691228 | Zaragoza, Spain | 2005 | *Chenopodium album* | C.a-2-05 | 41.63°N, 0.88°W |
| JQ691226 | Zaragoza, Spain | 2005 | *Solanum nigrum* | S.n-3-05 | 41.63°N, 0.88°W |
| JQ691225 | Zaragoza, Spain | 2005 | *Solanum nigrum* | S.n.-1-05 | 41.63°N, 0.88°W |
| JQ691224 | Zaragoza, Spain | 2006 | *Vicia faba* | Hb-2-06 | 41.63°N, 0.88°W |
| JQ691222 | Zaragoza, Spain | 2000 | *Borago officinalis* | Bo-1-00 | 41.63°N, 0.88°W |
| JQ691217 | Zaragoza, Spain | 2005 | *Solanum lycopersicum* | T-8-05 | 41.63°N, 0.88°W |
| JQ691214 | Zaragoza, Spain | 1992 | *Solanum lycopersicum* | T-4-92 | 41.63°N, 0.88°W |
| JQ691213 | Zaragoza, Spain | 1988 | *Solanum lycopersicum* | T-5-88 | 41.63°N, 0.88°W |
| JQ691212 | Zaragoza, Spain | 1984 | *Solanum lycopersicum* | T-3-84 | 41.63°N, 0.88°W |
| JQ691211 | Zaragoza, Spain | 2007 | *Capsicum annuum* | P-5-07 | 41.63°N, 0.88°W |
| JQ691208 | Zaragoza, Spain | 2002 | *Capsicum annuum* | P-46-02 | 41.63°N, 0.88°W |
| JQ691207 | Zaragoza, Spain | 1984 | *Capsicum annuum* | P-62-84 | 41.63°N, 0.88°W |
| JQ691206 | Zaragoza, Spain | 1984 | *Capsicum annuum* | P-38-84 | 41.63°N, 0.88°W |
| JQ691205 | Badajoz, Spain | 1982 | *Capsicum annuum* | P-31-82 | 38.86°N, 6.96°W |
| JQ691204 | Badajoz, Spain | 1981 | *Capsicum annuum* | P-36-81 | 38.86°N, 6.96°W |
| JQ691202 | Gerona, Spain | 2007 | *Medicago sativa* | Alf-19-07 | 41.15°N, 2.75°E |
| JQ691201 | Gerona, Spain | 2007 | *Medicago sativa* | Alf-17-07 | 41.15°N, 2.75°E |
| JQ691200 | Gerona, Spain | 2007 | *Medicago sativa* | Alf-8-07 | 41.15°N, 2.75°E |
| JQ691199 | Gerona, Spain | 2007 | *Medicago sativa* | Alf-7-07 | 41.15°N, 2.75°E |
| JQ691198 | Gerona, Spain | 2007 | *Medicago sativa* | Alf-6-07 | 41.15°N, 2.75°E |
| JQ691197 | Gerona, Spain | 2007 | *Medicago sativa* | Alf-5-07 | 41.15°N, 2.75°E |
| JQ691196 | Gerona, Spain | 2007 | *Medicago sativa* | Alf-4-07 | 41.15°N, 2.75°E |
| JQ691194 | Zaragoza, Spain | 2007 | *Medicago sativa* | Alf-2-07 | 41.63°N, 0.88°W |
| JQ691192 | Huesca, Spain | 2006 | *Medicago sativa* | Alf-4-06 | 42.13°N, 0.4°W |
| JQ691189 | Zaragoza, Spain | 2005 | *Medicago sativa* | Alf-39-05 | 41.63°N, 0.88°W |
| FR715042 | Almeria, Spain | 31-May-11 | *Tecomaria capensis* | Tec1 | 36.83°N, 2.45°W |

**Supplementary Table S3**. Chinese provinces in the different bioclimatic zones.

| **Bioclimatic zones** | **Chinese provinces with the collection sites** |
| --- | --- |
| Temperate continental climate | Yining, Xinjiang; Wuzhong, Ningxia; Wuzhong, Ningxia; Xilinhaote, Inner Mongolia; Lanzhou, Gansu; Urumqi, Xinjiang |
| Temperate monsoon climate | Yining, Xinjiang; Wuzhong, Ningxia; Wuzhong, Ningxia; Xilinhaote, Inner Mongolia; Lanzhou, Gansu; Urumqi, Xinjiang |
| Plateau mountain climate | Xianggelila, Yunnan; Wulan, Qinghai; Rikaze, Tibet; Lasa, Tibet; Xining, Qinghai; Minhe, Qinghai |
| Subtropical monsoon climate | Dali, Yunnan; Bengbu, Anhui; Lin’an, Zhejiang; Hangzhou, Zhejiang; Guiyang, Guizhou |
| Tropical monsoon climate |  |

**Supplementary Table S4.** Primers for amplification of AMV fragments by RT-PCR

| **Primer name** | **Sequence (5ʹ to 3ʹ)** |
| --- | --- |
| AMV-F | GTGCATTATCCACACGTAAGG |
| AMV-R | CATCACCGGAAGCGACAACG |

**Supplementary Table S5.** Primers for AMV-*cp* fragment by RT-PCR

| **Primer name** | **Sequence (5ʹ to 3ʹ)** |
| --- | --- |
| *cp*-F | ATGAGTTCTTCACAAAAGAAAGCTGGT |
| *cp*-R | ATGGGGTACATCAATGACGATCAAGATCG |

**Supplementary Table S6.** Sites under selection for Chinese AMV population (See the excel file)

| **Source of variation** | **d.f.** | **Sum of squares** | **Variance components** | **Percentage of variation** | **Wright’s statistics** | ***P*** |
| --- | --- | --- | --- | --- | --- | --- |
| Among provinces | 13 | 461.714 | 2.088 | 10.55 | *Φ_CT_* = 0.1055 | 0.0289 |
| Among localities | 10 | 204.881 | 1.024 | 5.17 | *Φ_SC_* = 0.0578 | 0.2408 |
| Within localities | 76 | 1267.923 | 16.683 | 84.28 | *Φ_ST_* = 0.1572 | 0.0001 |
| Total | 99 | 1934.518 | 19.795 |  |  |  |

**Supplementary Table S7.** Analysis of molecular variance for Chinese AMV populations for administrative divisions based on the provinces

| **Source of variation** | **d.f.** | **Sum of squares** | **Variance components** | **Percentage of variation** | **Wright’s statistics** | ***P*** |
| --- | --- | --- | --- | --- | --- | --- |
| Among climatic zones | 3 | 168.795 | 1.157 | 5.800 | *Φ_CT_* = 0.0580 | 0.0174 |
| Among localities | 23 | 553.787 | 2.193 | 10.991 | *Φ_SC_* = 0.1167 | 0.0045 |
| Within localities | 73 | 1211.936 | 16.602 | 83.208 | *Φ_ST_* = 0.1679 | 0.0003 |
| Total | 99 | 1934.518 | 19.952 |  |  |  |

**Supplementary Table S8.** Analysis of molecular variance for Chinese AMV populations based on the climatic zones

**Supplementary Table S9.** Analysis of molecular variance for Iranian AMV populations for administrative divisions based on the provinces

| **Source of variation** | **d.f.** | **Sum of squares** | **Variance components** | **Percentage of variation** | **Wright’s statistics** | ***P*** |
| --- | --- | --- | --- | --- | --- | --- |
| Among provinces | 15 | 227.055 | 0.886 | 4.68 | *Φ_CT_* = 0.0636 | 0.2313 |
| Among localities | 36 | 402.365 | 0 | 0 | *Φ_SC_* = −0.3837 | 0.9942 |
| Within localities | 19 | 342.833 | 18.044 | 95.32 | *Φ_ST_* = −0.2957 | 0.9545 |
| Total | 70 | 972.254 | 18.930 |  |  |  |

**Supplementary Table S10.** Sites under selection for Iranian AMV population (See the excel file)

**Supplementary Table S11.** Sites under selection for Spanish AMV population (See the excel file)

| **Source of variation** | **d.f.** | **Sum of squares** | **Variance components** | **Percentage of variation** | **Wright’s statistics** | ***P*** |
| --- | --- | --- | --- | --- | --- | --- |
| Among autonomies | 4 | 112.068 | 0 | 0 | *Φ_CT_* = −0.0799 | 0.5182 |
| Among provinces | 2 | 46.956 | 3.935 | 22.71 | *Φ_SC_* = 0.2271 | 0.1592 |
| Within provinces | 48 | 642.685 | 13.389 | 77.29 | *Φ_ST_* = 0.1654 | 0.0026 |
| Total | 54 | 801.709 | 17.324 |  |  |  |

**Supplementary Table S12**. Analysis of molecular variance for Spanish AMV populations for administrative divisions based on the autonomous regions

**Supplementary Table S13**. BaTS algorithm analysis for Spanish AMV population based on the host species level

|  | **Mean** | **Lower 95% HPD** | **Higher 95% HPD** | ***P*** |
| --- | --- | --- | --- | --- |
| **AI** | 4.8063 | 4.2064 | 5.3855 | 0.039 |
| **PS** | 28.8777 | 28 | 29 | 0.0010 |
| MC (*T. capensis*) |  |  |  |  |
| MC (*C. annuum*) | 1.9516 | 2 | 2 | 0.0620 |
| MC (*M. sativa*) | 2.7791 | 2 | 5 | 0.069 |
| MC (*N. tabacum*) |  |  |  |  |
| MC (*H. rosa-sinensis*) |  |  |  |  |
| MC (*S. lycopersicum*) |  |  |  |  |
| MC (*V. faba*) |  |  |  |  |
| MC(*S. nigrum*) |  |  |  |  |
| MC(*C. album*) |  |  |  |  |
| MC(*T. repens*) |  |  |  |  |
| MC(*C. sepium*) |  |  |  |  |
| MC(*S. oleraceus*) |  |  |  |  |
| MC(*P. echioides*) |  |  |  |  |
| MC(*M. sylvestris*) |  |  |  |  |

**Supplementary Table S14.** BaTS algorithm analysis for Spanish AMV population based on the host family level

|  | **Mean** | **Lower 95% HPD** | **Higher 95% HPD** | ***P*** | |
| --- | --- | --- | --- | --- | --- |
| **AI** | 3.77 | 3.1391 | 4.3625 | | 0.1850 |
| **PS** | 19.8318 | 19 | 20 | | 0.0030 |
| MC(*Bignoniaceae*) |  |  |  | |  |
| MC(*Solanaceae*) |  |  |  | |  |
| MC(*Fabaceae)* |  |  |  | |  |
| MC(*Malvaceae*) |  |  |  | |  |
| MC(*Chenopodiaceae*) |  |  |  | |  |
| MC(*Convulvulaceae*) |  |  |  | |  |
| MC(*Asteraceae*) |  |  |  | |  |

**Supplementary Table S15**. BaTS algorithm analysis for Iranian AMV population based on the host species level

|  | **Mean** | **Lower 95% HPD** | **Higher 95% HPD** | ***P*** |
| --- | --- | --- | --- | --- |
| **AI** | 5.6039 | 4.8627 | 6.3182 | 0.649 |
| **PS** | 28.6448 | 27 | 30 | 0.172 |
| MC(*M. sativa*) |  |  |  |  |
| MC(*D. carota*) |  |  |  |  |
| MC(*N. tabacum*) |  |  |  |  |
| MC(*S. tuberosum*) |  |  |  |  |
| MC(*C. album*) |  |  |  |  |
| MC(*S. asper*) |  |  |  |  |
| MC(*Trogopogon* sp.) |  |  |  |  |
| MC(*S. nigrum*) |  |  |  |  |
| MC(*V. unguiculata*) |  |  |  |  |
| MC(*C. frutescens*) |  |  |  |  |
| MC(*S. lycopersicum*) |  |  |  |  |
| MC(*C. annuum*) |  |  |  |  |
| MC(*A. majus*) |  |  |  |  |
| MC(*Rumex* sp.) |  |  |  |  |
| MC(*Plantago* sp.) |  |  |  |  |
| MC(*C. radicans*) |  |  |  |  |

|  | **Mean** | **Lower 95% HPD** | **Higher 95% HPD** | ***P*** | |
| --- | --- | --- | --- | --- | --- |
| **AI** | 5.2679 | 4.4692 | 6.0448 | | 0.8010 |
| **PS** | 26.7774 | 25 | 28 | | 0.4820 |
| MC (*Fabaceae*) |  |  |  | |  |
| MC (*Apiaceae*) |  |  |  | |  |
| MC (*Solanaceae*) |  |  |  | |  |
| MC (*Amaranthaceae*) |  |  |  | |  |
| MC (*Asteraceae*) |  |  |  | |  |
| MC (*Plantaginaceae*) |  |  |  | |  |
| MC (*Polygonaceae*) |  |  |  | |  |
| MC (*Bignoniaceae*) |  |  |  | |  |

**Supplementary Table S16.** BaTS algorithm analysis for Iranian AMV population based on the host family level
